# Postembolization Syndrome after Prostatic Artery Embolization: A Systematic Review

**DOI:** 10.3390/diagnostics10090659

**Published:** 2020-08-31

**Authors:** Petra Svarc, Mikkel Taudorf, Michael Bachmann Nielsen, Hein Vincent Stroomberg, Martin Andreas Røder, Lars Lönn

**Affiliations:** 1 Department of Radiology, Rigshospitalet, Faculty of Health and Medical Sciences, University of Copenhagen, Blegdamsvej 9, 2100 Copenhagen, Denmark; Mikkel.Taudorf@regionh.dk (M.T.); mbn@dadlnet.dk (M.B.N.); lars.birger.loenn@regionh.dk (L.L.); 2 Copenhagen Prostate Cancer Center, Department of Urology, Rigshospitalet, Faculty of Health and Medical Sciences, University of Copenhagen, Blegdamsvej 9, 2100 Copenhagen, Denmark; hein.vincent.stroomberg@regionh.dk (H.V.S.); Martin.Andreas.Roeder@regionh.dk (M.A.R.)

**Keywords:** prostatic artery embolization, benign prostatic hyperplasia, postembolization syndrome

## Abstract

Postembolization syndrome (PES) is the most common side effect of vascular embolization of solid organs. The aim of this review was to determine the incidence of PES and its individual components after prostatic artery embolization (PAE). A systematic review with a pre-specified search strategy for PubMed, Embase, Web of Science and Cochrane Library was performed according to PRISMA guidelines. Studies in English regarding PAE in humans with 10 or more participants were eligible for inclusion. No restrictions on participant demographics or PAE technique were imposed. The search returned 378 references, of which 32 studies with a total of 2116 patients met the inclusion criteria. The results for overall PES frequency and individual PES components were presented as median (interquartile range, (*IQR*)). Overall median PES frequency was 25.5% (12.5–45.8). The two most frequent individual PES components were dysuria/urethral burning and local pain, with a median frequency of 21.7% (13.8–33.3) and 20% (5.4–29.4), respectively. Most outcome measures were characterized by a marked lack of uniformity and inconsistency in reporting across studies. Development of a uniform reporting system would help the clinicians recognize and treat PES accordingly.

## 1. Introduction

Benign prostatic hyperplasia (BPH) is a frequent cause of lower urinary tract symptoms (LUTS) in men [1,2], with one fourth of men older than 70 years having moderate to severe LUTS that impair their quality of life (QOL) [3]. Prostatic artery embolization (PAE) is a new minimally invasive technique proven effective in reducing LUTS in BPH comparable to the preferred surgical treatment—the transurethral resection of the prostate (TURP) [4,5,6,7]. The most common side effect of vascular embolization of solid organs is a collection of inflammation- and tissue necrosis-related symptoms known as the postembolization syndrome (PES) [8,9,10]. The syndrome is characterized by influenza-like symptoms, pain and nausea and, in the case of PAE, dysuria and transient worsening of LUTS. Leukocytosis, leukopenia and/or elevation of C-reactive protein are also commonly seen [11]. The symptoms vary in their severity and duration and can, if pronounced, be mistaken for urosepsis. Consequently, a subset of patients may need admission to hospital for observation and symptomatic treatment, increasing the overall procedural costs. No uniform system for reporting PES exists, making its incidence fluctuate widely between studies. Moreover, trials investigating postoperative management plans or drugs to reduce PES do not exist, and PES is currently treated symptomatically with a combination of analgesics, antipyretics and antiemetics. No dedicated systematic reviews examining all components of PES after PAE have been published to date. Thus, there is a lack of deeper insight into incidence, grade and future management of PES after PAE. The aim of this study was to determine the incidence of PES and its components after PAE and subsequently assist the clinicians in correctly recognizing and treating the syndrome.

## 2. Materials and Methods

### 2.1. Protocol and Registration

This systematic review was conducted in accordance to the Preferred Reporting Items for Systematic Review and Metanalysis (PRISMA) guidelines [12], and a published protocol with pre-specified inclusion criteria, outcomes and search strategy can be found in the PROSPERO database (PROSPERO ID: CRD42020164472) [13].

### 2.2. Information Sources and Search Strategy

PubMed, Embase, Web of Science and Cochrane Library were searched. The following search terms were applied: benign AND prostat* AND (hyperplasia OR hypertrophy OR enlargement OR obstruction) AND emboli?ation AND ALL= (side?effect* OR complication* OR adverse effect*). MeSH terms used were “Embolization, Therapeutic” and “Prostatic Hyperplasia”. The search terms were combined and conducted in appropriate combinations on 16 January 2020. A new search conducted on 1 June 2020 returned no new studies eligible for inclusion.

### 2.3. Eligibility Criteria and Study Selection

Studies regarding PAE in humans with 10 or more subjects were eligible for inclusion. Reviews, case reports, abstracts, supplements and conference papers as well as articles not published in English were excluded. No restrictions on publication dates were imposed. Two authors (P.S. and M.T.) reviewed abstracts. Full text of all included articles was obtained and read by the same two authors. Agreement was reached through consensus using Covidence Systematic Review software (Veritas Health Innovation, Melbourne, Australia) [14]. First author, publication year, study location, data collection period, study design, number of patients and outcome measures for all included articles were collected. Outcome measures were extracted in duplicate in a piloted data-extraction form.

### 2.4. Outcome Measures

The primary outcome measure was the overall percentage of PES in studies selected for the review. The secondary outcome measures were the overall percentages of each individual PES component. In the context of this review, PES was defined as one or more of the following components: fever, local (perineal, retroperitoneal, pelvic, perianal, urethral or retropubic) pain, nausea with or without vomiting, dysuria/urethral burning and transient worsening of LUTS. If an article reported separately more than one of the above PES components, and it was unclear if a single patient experienced more than one symptom, the component with the highest reported percentage was taken to represent the overall PES percentage in the study. In articles not reporting one or more of the above outcomes, that outcome is presumed not to have been recorded and not as having not occurred.

### 2.5. Risk of Bias

Risk of bias in randomized trials (RCTs) was assessed using the Cochrane Risk of Bias tool (RoB 2.0) [15]. Non-randomized trials were assessed for risk of bias using the Risk of Bias In Non-randomized Studies-of Interventions (ROBINS-I) tool [16]. Robvis online visualization tool was used to graphically present the risk of bias data [17].

### 2.6. Statistical Considerations

Due to study heterogeneity meta-analysis was not possible. The outcomes are presented as median (interquartile range, (*IQR*)).

## 3. Results

### 3.1. Study Selection and Overview

**Figure 1:**
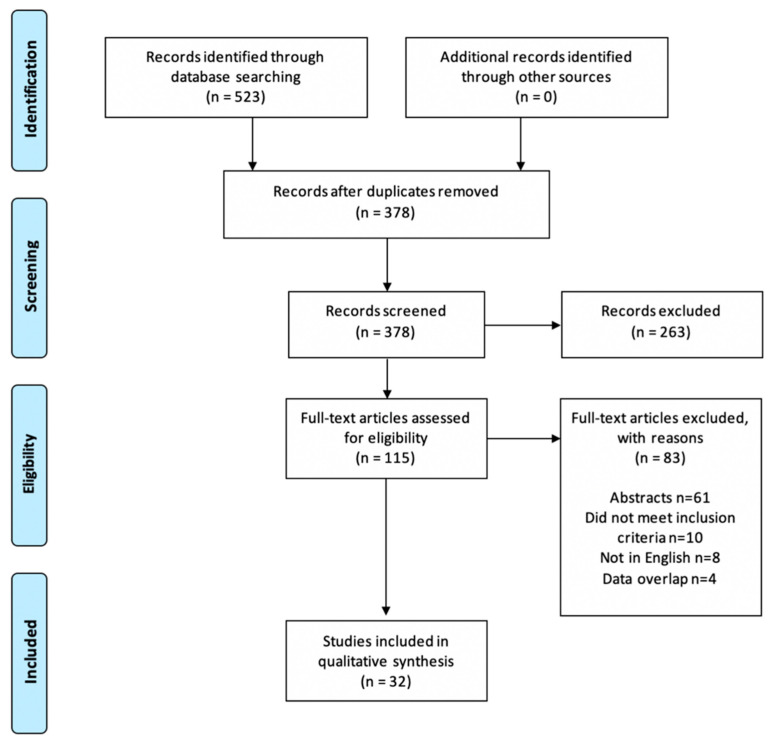
PRISMA flow diagram.

The database search returned 378 references with duplicates removed. A total of 263 articles were removed after reading the abstract. Of the remaining 115 studies assessed for full-text eligibility, 32 studies with a total of 2116 patients (ranging from 11–199) were selected for data extraction [5,6,7,18,19,20,21,22,23,24,25,26,27,28,29,30,31,32,33,34,35,36,37,38,39,40,41,42,43,44,45,46]. A PRISMA flow diagram depicts the process of study selection (Figure 1).

Seven of the included studies were RCTs [5,6,7,22,23,24,25], as presented in Table 1. Study characteristics of prospective and retrospective studies are presented in Table 2 and Table 3, respectively.

### 3.2. Outcome Measures

**Figure 2:**
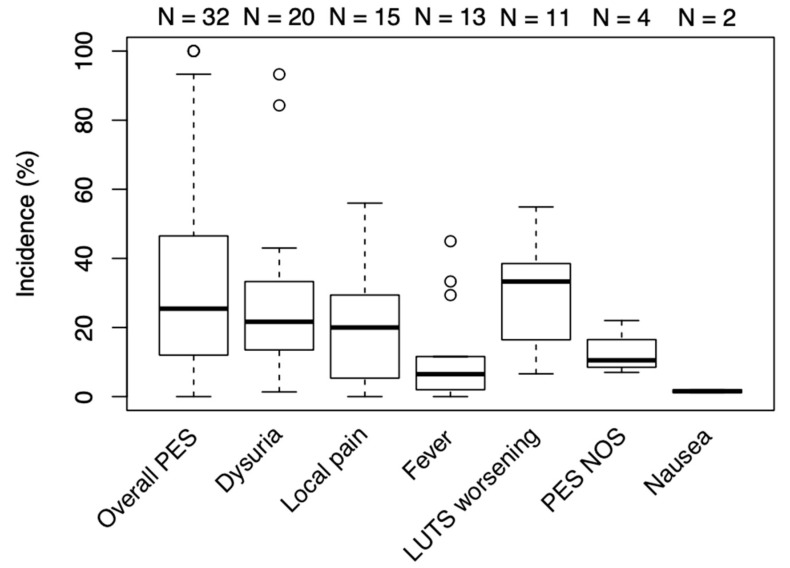
Median frequency of PES and its components. Box = 25th and 75th percentiles; bars = minimum and maximum values (1.5× IQR); bold line = median; N = number of studies included; outliers represented as circles. LUTS, lower urinary tract symptoms; PES, postembolization syndrome; NOS, not otherwise specified.

The most frequently reported symptom was dysuria or urethral burning, appearing in 20 of the 32 included studies. Local pain and fever were the second and third-most reported symptoms, present in 16 and 13 studies, respectively. The most infrequently mentioned symptom was nausea, appearing in just 2 studies. Four studies mentioned PES but did not provide their definition of the syndrome. Four studies had PES explicitly defined and recorded as a collection of symptoms, but no studies mentioned all the PES components as defined in outcome measures. Overlap between symptoms and patients was difficult to determine in studies where several PES components were mentioned independently. The overall median PES percentage was 25.5% (12.5–45.8), and median percentages of the individual PES components were as follows: 33.3% (16.5–38.5) for LUTS worsening, 21.7% (13.8–33.3) for dysuria/urethral burning, 20% (5.4–29.4) for local pain, 6.5% (2–11.6) for fever and 1.6% (1.3–18) for nausea and/or vomiting. The data with outliers is presented as a box plot in Figure 2.

Two studies [6,20] reported the overall PES frequency to be 100%. The highest reported percentages for individual PES components were 93% for dysuria and/or urethral burning [33], 56% for local pain [7], 54% for LUTS worsening [30], 45% for fever [25] and 2% for nausea [30]. Symptoms with the most pronounced lack of uniformity in reporting were overall PES (ranging from 0% to 100%), urethral burning and/or dysuria (ranging from 1.35% to 93.3%) and fever (ranging from 0% to 45%). Remaining outcome measures had a more uniform distribution, with all data points within the 1.5× IQR from the first and third quartiles.

### 3.3. Risk of Bias

**Figure 3:**
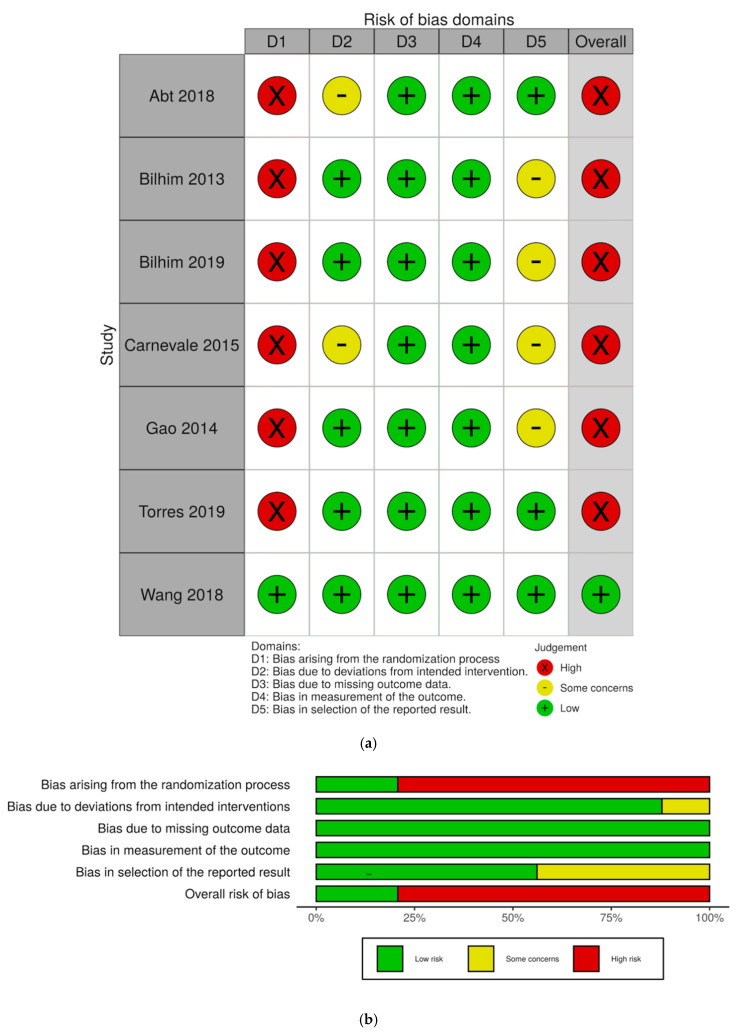
RoB2 assessments of RCTs. (**a**) “Traffic-light” illustration of risk of bias in individual studies. (**b**) Weighted bar plots depicting risk of bias judgement distributions within each domain.

**Figure A1:**
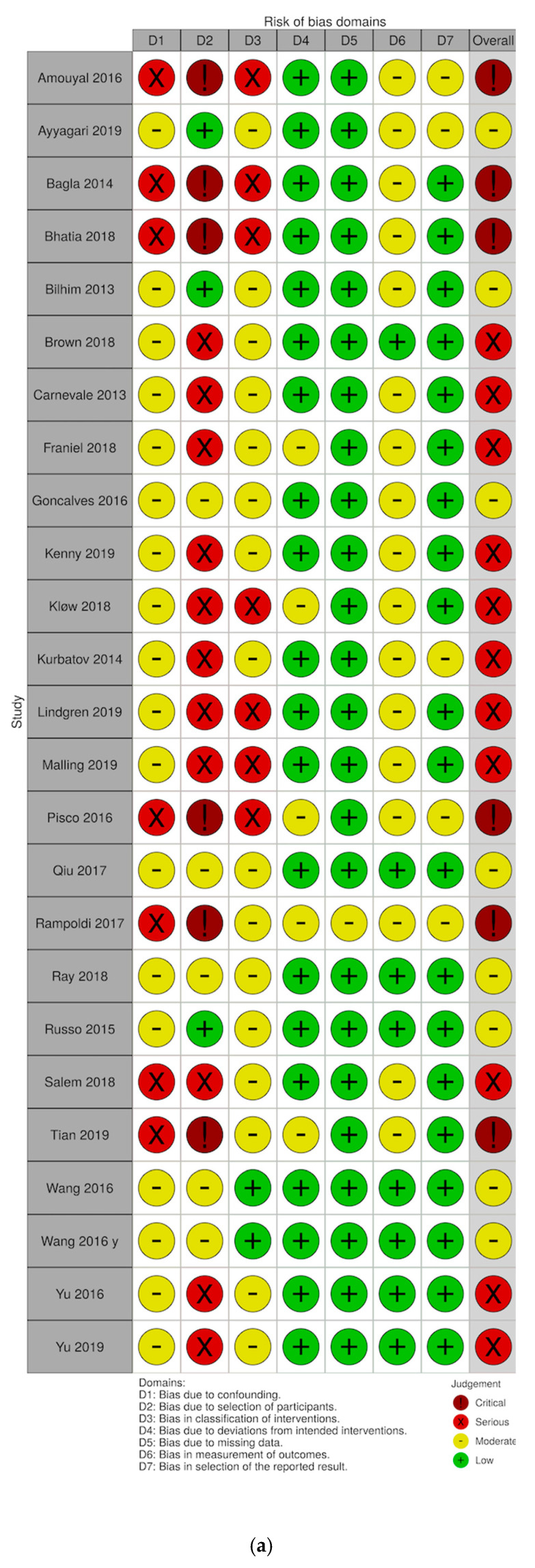

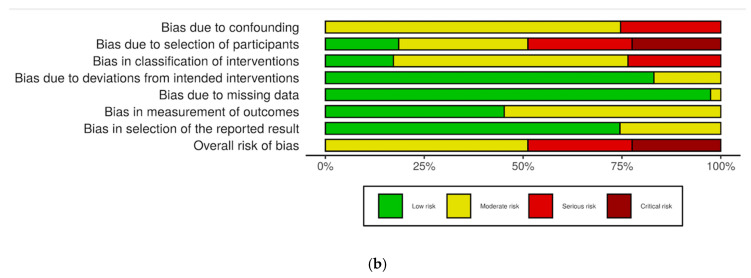
Risk of bias in non-randomized studies-of interventions (ROBINS-I) assessments of non-RCTs. (**a**) “Traffic-light” illustration of risk of bias in individual studies. (**b**) Weighted bar plots depicting risk of bias judgement distributions within each domain.

Risk of bias assessments and judgement distribution within each domain for the randomized studies are visualized as “traffic-light” and weighted bar plots using the robvis tool [17] (Figure 3a,b). The most common risk of bias in the RCTs was bias due to randomization process, making all but one study at a high risk of bias. Likewise, most of the non-randomized studies were assessed to be at either moderate or high risk of bias, owing to their retrospective design and lack of control groups (Figure A1).

## 4. Discussion

This systematic review is the first dedicated review investigating the overall incidence and individual components of PES after PAE. From the data of 32 studies with a total of 2116 patients, we have demonstrated an overall median PES incidence of 25.5% with a pronounced lack of uniformity in reporting between studies.

PAE is a procedure that can reduce LUTS in men with BPH, demonstrated to be safe and rarely associated with severe complications, such as non-target embolization. PES is a well-known side effect of endovascular arterial embolization in other organs or tumors. However, PES is often overlooked when reporting the possible side effects to PAE, and no consensus exists on whether it is an expected side effect to PAE or a complication to the procedure, even though PES may temporarily impair quality of life and lead to secondary hospital admissions for pain and/or fever management. This review has underlined that PES is indeed very common. Surprisingly, no uniform reporting of PES exists, which raises concerns about its true frequency following PAE.

Several studies have addressed the pathogenesis and incidence of PES in other organs [10,11]. In PAE, Moreira et al. [8] were one of the first to describe PES as the most common side-effect of PAE. The symptoms of PES are typically followed by leucopenia, leukocytosis and/or elevation of C-reactive protein (CRP) [9,10,11], which suggests that systemic manifestations of PES (fever, nausea, malaise) could be regarded as components of the systemic inflammatory response syndrome (SIRS) [47]. This is most likely caused by prostate tissue hypoxia and cell death mediated release of tissue breakdown products, inflammatory mediators (interleukin-6, tumor necrosis factor α, and others) and vasoactive substances [11]. Similarly, periprostatic and prostatic inflammatory response is probably responsible for observed local PES components (local pain, dysuria and LUTS worsening) [8]. The prostate is innervated with an abundant nervous complex that ultimately ends in the corpora cavernosa. Most nerves are noradrenergic fibers that via alfa-1-adrenoreceptors cause smooth muscle contraction. It is likely that ischemia and necrosis activate nervous innervation and lead to frequent urination and urgency. The release of inflammatory mediators may be responsible for the pain observed by men with PES. It is well-known from bacterial and non-bacterial prostatitis that inflammation of the prostate results in diffuse pain in the pelvis area, tip of the penis and dysuria. It is striking that the severity of PES varies widely between patients. Wang et al. [43] showed that large size prostates (>80 cm^3^) had a statistically significant increase in risk for urethral burning compared to smaller prostates (16.7% vs 10.2% for urethral burning, respectively), suggesting a proportional relationship between prostate size and symptom severity.

Reported incidence of PES in other anatomical sites varies from 40% in uterine artery embolization [11] to 89% in renal angiomyolipoma embolization [10]. Empirical observations from our own group of men undergoing PAE suggest that PES occurs in up to 90% with a varying degree of severity ranging from admission to hospital to only mild discomfort 2–3 days after intervention. In contrast, the median overall PES incidence in this review was only 25.5%. The incidence ranged from 0% in studies by Kurbatov et al. [18] and Yu et al. [45] to a 100% in an RCT conducted by Carnevale et al. [6] and a study by Amouyal et al. [20]. This underreporting of PES symptoms in some studies can partially be explained by a stance held by some authors that PES symptoms are not to be regarded as complications but as expected neglectable side-effects to PAE and are consequently not mentioned in publications [48]. Additionally, the overall PES incidence in this review probably underestimated the true overall figure due to unclear overlap between patients and symptoms in 19 of the 32 studies, resulting in an inability to combine different individual PES components.

PES is a self-limiting condition that is treated symptomatically with a combination of analgesics, antiemetics and antipyretics. However, PES can be so severe that patients experience high fever, shivers, dysuria and urgency mimicking a septicemia from the urinary tract. As shown by Ganguli et al. [11] in uterine artery embolization, leukocytosis is frequent after solid organ embolization, further complicating the discerption of PES from infection. In this review, the incidence of urinary tract infections (UTIs) requiring antibiotic treatment as reported by 20 studies was 2.7% (SD 3.7). Seven studies recorded no UTIs and the highest UTI percentage of 13.8% was reported in a study by Kløw et al. [35]. Currently, antibiotic prophylaxis covering Gram-negative rods is routinely administered prior to PAE in most centers, even though no randomized trials evaluating its efficacy exist to date. A study by Cochran et al. [49] regarding percutaneous nephrostomy tube placement found no significant difference in urosepsis rates in low-risk group with and without antibiotic prophylaxis, though reservations for small sample size had to be made. However, the same trial showed a significant decrease in urosepsis rates (from 50% to 9%) with antibiotic prophylaxis in high-risk group (advanced age, diabetes, bladder dysfunction, indwelling catheter, earlier manipulation, urointestinal anastomosis, bacteriuria and stones). This might suggest a more individual approach is needed in the future, especially in low-risk patients without significant comorbidities.

Following the inflammation hypothesis, prophylactic corticosteroids were used and proven successful in reducing the incidence, severity and duration of PES after renal angiomyolipoma ablation [10], endovascular abdominal aortic repair (EVAR) [50] and transcatheter arterial chemoembolization (TACE) of the liver [51]. The last two studies were conducted as double-blind randomized placebo-controlled trials with a low risk of bias, providing good evidence quality for corticosteroid usage. Administration of a single-dose perioperative corticosteroid was not associated with any significant side-effects in a meta-analysis of RCTs by De Oliveira et al. [52]. No similar studies were conducted concerning PES after PAE, and symptomatic therapy is still the mainstay treatment.

We believe that raised awareness and uniform reporting of incidence and symptoms of PES would help the clinicians recognize the syndrome correctly, avoiding unnecessary antibiotics treatment and hospital admission. Patient information on the symptoms of PES is also crucial to optimize care. We suggest that the presence of dysuria, urgency, frequent urination, nausea, fever, pelvis or prostate pain, urine retention or overall worsening of LUTS during the first 7 days following PAE be regarded and reported as PES no matter if they occur individually or together. This would greatly improve the transparency and uniformity of reporting in future publications. Moreover, we urge the PAE community to address PES in interventional trials in order to reduce the incidence and/or duration of PES following PAE.

This systematic review is limited by heterogeneity in patient inclusion and exclusion criteria across studies as well as the use of different embolization techniques and material. This resulted in a heterogeneous group of studies with no possibility for meta-analysis. Additionally, overall PES frequency was probably underestimated due to underreporting as well as difficulties in calculating overall PES frequency from individual PES components.

## 5. Conclusions

PES is the most frequent adverse event following PAE. This systematic review showed a lack of uniformity in reporting the symptoms of PES after PAE. We urge the PAE community to define the criteria for PES to improve transparency and help the clinicians recognize and treat the symptoms accordingly. Further studies to reduce PES after PAE are also warranted.

## Figures and Tables

**Table 1:** Study characteristics of randomized trials (RCTs).

Author and (Year)	Study Design	Data Collection Period	Study Location	Patients Included in Intervention Group(s) (*n*)	Mean Age	Intervention	Control/Comparator
Abt (2018) [7]	open-label RCT	Feb 2014–May 2017	Switzerland	48	65.7	PAE with 250–400 µm Embozene^®^	TURP
Bilhim (2013) [22]	single-blind RCT	May 2011–Dec 2011	Portugal	80	63.9	PAE with 80–180 µm or 180–300 µm particles	
Bilhim (2019) [23]	single-blind RCT	Nov 2017–Nov 2018	Portugal	84	67.3 cPAE; 65.8 bPAE	cPAE, bPAE (both with with 300–500 µm Embosphere^®^)	
Carnevale (2016) [6]	open-label RCT	Nov 2010–Dec 2012	Brazil	15	60.4	PAE PErFecTED with 300–500 µm Embosphere^®^	original PAE and TURP
Gao (2014) [5]	open-label RCT	Jan 2007–Jan 2012	China	54	67.7	PAE with 355–500 µm Ivalon^®^	TURP
Torres (2019) [24]	open-label RCT	Jul 2015–Dec 2016	Portugal	137	66.1	PAE (3 groups: 100–300 µm, 300–500 µm, and 100–300 followed by 300–500 µm microspheres)	
Wang (2018) [25]	double-blind RCT	Jan 2010–Oct 2015	China	110	69.5	PAE (2 groups: 50 µm followed by 100 µm and 100 µm spheres alone)	

*bPAE*, balloon-occlusion prostatic artery embolization; *cPAE*, conventional microcatheter prostatic artery embolization; *PAE*, prostatic artery embolization; *PErFecTED*, proximal embolization first then embolize distant; *TURP*, transurethral resection of the prostate.

**Table 2:** Study characteristics of prospective studies.

Author and (Year)	Study Design	Data Collection Period	Study Location	Patients Included in Intervention Group(s) (*n*)	Mean Age	Intervention	Control/Comparator
Bagla (2014) [27]	prospective	Jan 2012–Mar 2013	United States	19	66.5	PAE with 100–400 µm Embozene^®^	
Bilhim (2013) [29]	prospective	Mar 2009–Dec 2011	Portugal	122	65.8 bilateral PAE; 71.3 unilateral PAE	PAE with 100- and 200 µm particle sizes, unilateral vs. bilateral	
Brown (2018) [30]	prospective	Nov 2015–Feb 2017	Australia	51	67	PAE with 250 µm Embozene^®^	
Carnevale (2013) [31]	prospective	Jun 2008–Nov 2011	Brazil	11	68.5	PAE with 300–500 µm Embosphere^®^	
Franiel (2018) [32]	prospective	Jul 2014–Dec 2015	Germany	27	66	PAE with 250 µm Embozene^®^	
Goncalves (2016) [33]	prospective	Aug 2011–Jun 2013	Brazil	30	not mentioned	PAE with 100–300 or 300–500 µm Embosphere^®^	
Kenny (2019) [34]	prospective	Not mentioned	France	20	75.3	PAE with 300–500 µm Bead Block^®^ in patients with indwelling catheters	
Kløw (2018) [35]	prospective	Dec 2015–Mar 2017	Norway	29	69	PAE with 300–500 µm Embosphere^®^	
Kurbatov (2014) [18]	prospective	Jan 2009–Jan 2012	Russia and Italy	88	66.4	PAE with 300–500 µm Embosphere^®^ in prostates >80 cm^3^	
Lindgren (2019) [36]	prospective	Jan 2015–Jun 2018	Sweden	37	73	PAE with 300–500 µm Embosphere^®^	
Malling (2019) [21]	prospective	Jul 2017–Jul 2018	Denmark	11	75.2	PAE PErFecTED with 300–500 µm Embosphere^®^	
Rampoldi (2017) [19]	prospective	Not mentioned	Italy	41	77.9	PAE PErFecTED with 300–500 µm Embosphere^®^ in patients with indwelling catheters	Indwelling urinary catheter
Ray (2018) [39]	prospective	Jul 2014–Jan 2016	United Kingdom	199	66	PAE	TURP
Russo (2015) [40]	prospective matched pair	Jan 2006–Jan 2014	Italy	80	67	PAE with 300–500 µm Embosphere^®^	open prostatectomy
Salem (2018) [41]	prospective	Dec 2014–Jun 2017	United States	45	67	PAE with 300–500 µm Embosphere^®^	
Wang (2016) [43]	prospective	Apr 2010–Dec 2013	China	115	72.5 (>80 cm^3^); 66 (50–80 cm^3^)	PAE with 100 µm particles in prostates >80 cm^3^ and 50–80 cm^3^	
Wang (2016) [44]	prospective	Feb 2009–Apr 2014	China	158	82.5 (>75 yrs), 67.5 (<75 yrs)	PAE with 100 µm particles in men >75 years and <75 years	
Yu (2016) [45]	prospective	Jun 2015–Mar 2016	Hong Kong SAR	16	66	PAE with 100–300 µm Embosphere^®^ in patients with BPH and acute urinary retention	PAE with 100–300 µm Embosphere^®^ *n* patients with BPH without urinary retention
Yu (2019) [46]	prospective	Jun 2015–Dec 2018	Hong Kong SAR	82	66	PAE with 100–300 µm Embosphere^®^	

*BPH*, benign prostatic hyperplasia; *PAE*, prostatic artery embolization; *PErFecTED*, proximal embolization first then embolize distant; *TURP*, transurethral resection of the prostate.

**Table 3:** Study characteristics of retrospective studies.

Author and (Year)	Study Design	Data Collection Period	Study Location	Patients Included in Intervention group(s) (*n*)	Mean Age	Intervention	Control/Comparator
Amouyal (2016) [20]	retrospective	Dec 2013–Jan 2015	France	32	65	PAE PErFecTED with 300–500 µm Embosphere^®^	
Ayyagari (2019) [26]	retrospective	Apr 2013–Aug 2018	United States	93	76.0 end-hole; 72,8 balloon occlusion	end-hole vs. balloon occlusion PAE (both with 100–300 µm Embosphere^®^)	
Bhatia (2018) [28]	retrospective	Apr 2014–Oct 2017	United States	93	68.5	PAE with 100–300 or 300–500 µm Embosphere^®^	
Pisco (2016) [37]	retrospective	Mar 2009–Sep 2014	Portugal	152	67.4	PAE 100–200 µm PVA spheres, 300–500 µm Bead Block^®^, 300–500 µm Embosphere^®^ or 400 µm Embozene^®^	
Qiu (2017) [38]	retrospective	Feb 2012–Mar 2015	China	17	75.53	PAE with 90–180 µm Embosphere^®^	TURP
Tian (2019) [42]	retrospective	Feb 2014–Dec 2017	China	20	80.8	PAE with 90–180 µm or 180–300 µm particles for control of gross haematuria in BPH	

*BPH*, benign prostatic hyperplasia; *PAE*, prostatic artery embolization; *PErFecTED*, proximal embolization first then embolize distant; *TURP*, transurethral resection of the prostate.

## References

[1] Roehrborn C.G. (2005). Benign prostatic hyperplasia: An overview. Rev. Urol..

[2] Thorpe A., Neal D. (2003). Benign prostatic hyperplasia. Lancet.

[3] Irwin D.E., Kopp Z.S., Agatep B., Milsom I., Abrams P. (2011). Worldwide prevalence estimates of lower urinary tract symptoms, overactive bladder, urinary incontinence and bladder outlet obstruction: Worldwide prevalence of luts. BJU Int..

[4] Malling B., Røder M.A., Brasso K., Forman J., Taudorf M., Lönn L. (2019). Prostate artery embolisation for benign prostatic hyperplasia: A systematic review and meta-analysis. Eur. Radiol..

[5] Gao Y., Huang Y., Zhang R., Yang Y., Zhang Q., Hou M., Wang Y. (2014). Benign Prostatic Hyperplasia: Prostatic Arterial Embolization versus Transurethral Resection of the Prostate—A Prospective, Randomized, and Controlled Clinical Trial. Radiology.

[6] Carnevale F.C., Iscaife A., Yoshinaga E.M., Moreira A.M., Antunes A.A., Srougi M. (2016). Transurethral Resection of the Prostate (TURP) Versus Original and PErFecTED Prostate Artery Embolization (PAE) Due to Benign Prostatic Hyperplasia (BPH): Preliminary Results of a Single Center, Prospective, Urodynamic-Controlled Analysis. Cardiovasc. Interv. Radiol..

[7] Abt D., Hechelhammer L., Müllhaupt G., Markart S., Güsewell S., Kessler T.M., Schmid H.-P., Engeler D.S., Mordasini L. (2018). Comparison of prostatic artery embolisation (PAE) versus transurethral resection of the prostate (TURP) for benign prostatic hyperplasia: Randomised, open label, non-inferiority trial. BMJ.

[8] Moreira A.M., de Assis A.M., Carnevale F.C., Antunes A.A., Srougi M., Cerri G.G. (2017). A Review of Adverse Events Related to Prostatic Artery Embolization for Treatment of Bladder Outlet Obstruction Due to BPH. Cardiovasc. Interv. Radiol..

[9] Leung D.A., Goin J.E., Sickles C., Raskay B.J., Soulen M.C. (2001). Determinants of postembolization syndrome after hepatic chemoembolization. J. Vasc. Interv. Radiol..

[10] Bissler J.J., Racadio J., Donnelly L.F., Johnson N.D. (2002). Reduction of postembolization syndrome after ablation of renal angiomyolipoma. Am. J. Kidney Dis..

[11] Ganguli S., Faintuch S., Salazar G.M., Rabkin D.J. (2008). Postembolization Syndrome: Changes in White Blood Cell Counts Immediately after Uterine Artery Embolization. J. Vasc. Interv. Radiol..

[12] Moher D., Liberati A., Tetzlaff J., Altman D.G. (2009). The PRISMA Group. Preferred Reporting Items for Systematic Reviews and Meta-Analyses: The PRISMA Statement. PLoS Med..

[13] Svarc Petra Components and Incidence of the Postembolization Syndrome after Prostatic Artery Embolization for Benign Prostatic Hyperplasia: A Systematic Review. https://www.crd.york.ac.uk/PROSPERO/display_record.php?RecordID=164472.

[14] Covidence Systematic Review Software, Veritas Health Innovation, Melbourne, Australia. https://www.covidence.org.

[15] Sterne J.A.C., Savović J., Page M.J., Elbers R.G., Blencowe N.S., Boutron I., Cates C.J., Cheng H.-Y., Corbett M.S., Eldridge S.M. (2019). RoB 2: A revised tool for assessing risk of bias in randomised trials. BMJ.

[16] Sterne J.A., Hernán M.A., Reeves B.C., Savović J., Berkman N.D., Viswanathan M., Henry D., Altman D.G., Ansari M.T., Boutron I. (2016). ROBINS-I: A tool for assessing risk of bias in non-randomised studies of interventions. BMJ.

[17] McGuinness L.A., Higgins J.P.T. (2020). Risk-of-bias VISualisation (robvis): An R package and Shiny web app for visualising risk-of-bias assessments. Res. Synth. Methods.

[18] Kurbatov D., Russo G.I., Lepetukhin A., Dubsky S., Sitkin I., Morgia G., Rozhivanov R., Cimino S., Sansalone S. (2014). Prostatic Artery Embolization for Prostate Volume Greater Than 80 cm^3^: Results From a Single-center Prospective Study. Urology.

[19] Rampoldi A., Barbosa F., Secco S., Migliorisi C., Galfano A., Prestini G., Harward S.H., Di Trapani D., Brambillasca P.M., Ruggero V. (2017). Prostatic Artery Embolization as an Alternative to Indwelling Bladder Catheterization to Manage Benign Prostatic Hyperplasia in Poor Surgical Candidates. Cardiovasc. Interv. Radiol..

[20] Amouyal G., Thiounn N., Pellerin O., Yen-Ting L., Del Giudice C., Dean C., Pereira H., Chatellier G., Sapoval M. (2016). Clinical Results After Prostatic Artery Embolization Using the PErFecTED Technique: A Single-Center Study. Cardiovasc. Interv. Radiol..

[21] (2019). Malling; Lönn; Jensen; Lindh; Frevert; Brasso; Røder Prostate Artery Embolization for Lower Urinary Tract Symptoms in Men Unfit for Surgery. Diagnostics.

[22] Bilhim T., Pisco J., Campos Pinheiro L., Rio Tinto H., Fernandes L., Pereira J.A., Duarte M., Oliveira A.G. (2013). Does Polyvinyl Alcohol Particle Size Change the Outcome of Prostatic Arterial Embolization for Benign Prostatic Hyperplasia? Results from a Single-Center Randomized Prospective Study. J. Vasc. Interv. Radiol..

[23] Bilhim T., Costa N.V., Torres D., Pisco J., Carmo S., Oliveira A.G. (2019). Randomized Clinical Trial of Balloon Occlusion versus Conventional Microcatheter Prostatic Artery Embolization for Benign Prostatic Hyperplasia. J. Vasc. Interv. Radiol..

[24] Torres D., Costa N.V., Pisco J., Pinheiro L.C., Oliveira A.G., Bilhim T. (2019). Prostatic Artery Embolization for Benign Prostatic Hyperplasia: Prospective Randomized Trial of 100–300 μm versus 300–500 μm versus 100- to 300-μm + 300- to 500-μm Embospheres. J. Vasc. Interv. Radiol..

[25] Wang M.Q., Zhang J.L., Xin H.N., Yuan K., Yan J., Wang Y., Zhang G.D., Fu J.X. (2018). Comparison of Clinical Outcomes of Prostatic Artery Embolization with 50-μm Plus 100-μm Polyvinyl Alcohol (PVA) Particles versus 100-μm PVA Particles Alone: A Prospective Randomized Trial. J. Vasc. Interv. Radiol..

[26] Ayyagari R., Powell T., Staib L., Chapiro J., Schoenberger S., Devito R., Pollak J. (2019). Case-Control Comparison of Conventional End-Hole versus Balloon-Occlusion Microcatheter Prostatic Artery Embolization for Treatment of Symptomatic Benign Prostatic Hyperplasia. J. Vasc. Interv. Radiol..

[27] Bagla S., Martin C.P., van Breda A., Sheridan M.J., Sterling K.M., Papadouris D., Rholl K.S., Smirniotopoulos J.B., van Breda A. (2014). Early Results from a United States Trial of Prostatic Artery Embolization in the Treatment of Benign Prostatic Hyperplasia. J. Vasc. Interv. Radiol..

[28] Bhatia S., Sinha V.K., Harward S., Gomez C., Kava B.R., Parekh D.J. (2018). Prostate Artery Embolization in Patients with Prostate Volumes of 80 mL or More: A Single-Institution Retrospective Experience of 93 Patients. J. Vasc. Interv. Radiol..

[29] Bilhim T., Pisco J., Rio Tinto H., Fernandes L., Campos Pinheiro L., Duarte M., Pereira J.A., Oliveira A.G., O’Neill J. (2013). Unilateral Versus Bilateral Prostatic Arterial Embolization for Lower Urinary Tract Symptoms in Patients with Prostate Enlargement. Cardiovasc. Interv. Radiol..

[30] Brown N., Walker D., McBean R., Pokorny M., Kua B., Gianduzzo T., Dunglison N., Esler R., Yaxley J. (2018). Prostate artery Embolisation Assessment of Safety and feasibilitY (P-EASY): A potential alternative to long-term medical therapy for benign prostate hyperplasia. BJU Int..

[31] Carnevale F.C., da Motta-Leal-Filho J.M., Antunes A.A., Baroni R.H., Marcelino A.S.Z., Cerri L.M.O., Yoshinaga E.M., Cerri G.G., Srougi M. (2013). Quality of Life and Clinical Symptom Improvement Support Prostatic Artery Embolization for Patients with Acute Urinary Retention Caused by Benign Prostatic Hyperplasia. J. Vasc. Interv. Radiol..

[32] Franiel T., Aschenbach R., Trupp S., Lehmann T., von Rundstedt F.-C., Grimm M.-O., Teichgräber U. (2018). Prostatic Artery Embolization with 250-μm Spherical Polyzene-Coated Hydrogel Microspheres for Lower Urinary Tract Symptoms with Follow-up MR Imaging. J. Vasc. Interv. Radiol..

[33] Gonçalves O.M., Carnevale F.C., Moreira A.M., Antunes A.A., Rodrigues V.C., Srougi M. (2016). Comparative Study Using 100–300 Versus 300–500 μm Microspheres for Symptomatic Patients Due to Enlarged-BPH Prostates. Cardiovasc. Interv. Radiol..

[34] Kenny A.G., Pellerin O., Amouyal G., Desgranchamps F., Méria P., De Gouvello A., Dariane C., Déan C., Pereira H., Thiounn N. (2019). Prostate Artery Embolization in Patients With Acute Urinary Retention. Am. J. Med..

[35] Kløw N.E., Grøtta O.J., Bay D., Sandbæk G., Johansen T.E.B., Hagen T., Baco E. (2019). Outcome after prostatic artery embolization in patients with symptomatic benign prostatic hyperplasia. Acta Radiol..

[36] Lindgren H., Bläckberg M. (2019). Introduction of prostate artery embolization (PAE) in Sweden. Scand. J. Urol..

[37] Pisco J., Bilhim T., Pinheiro L.C., Fernandes L., Pereira J., Costa N.V., Duarte M., Oliveira A.G. (2016). Prostate Embolization as an Alternative to Open Surgery in Patients with Large Prostate and Moderate to Severe Lower Urinary Tract Symptoms. J. Vasc. Interv. Radiol..

[38] Qiu Z., Zhang C., Wang X., Cheng K., Liang X., Wang D., Hou S. (2017). Clinical evaluation of embolization of the superior vesical prostatic artery for treatment of benign prostatic hyperplasia: A single-center retrospective study. Videosurgery Other Miniinvasive Tech..

[39] Ray A.F., Powell J., Speakman M.J., Longford N.T., DasGupta R., Bryant T., Modi S., Dyer J., Harris M., Carolan-Rees G. (2018). Efficacy and safety of prostate artery embolization for benign prostatic hyperplasia: An observational study and propensity-matched comparison with transurethral resection of the prostate (the UK-ROPE study). BJU Int..

[40] Russo G.I., Kurbatov D., Sansalone S., Lepetukhin A., Dubsky S., Sitkin I., Salamone C., Fiorino L., Rozhivanov R., Cimino S. (2015). Prostatic Arterial Embolization vs Open Prostatectomy: A 1-Year Matched-pair Analysis of Functional Outcomes and Morbidities. Urology.

[41] Salem R., Hairston J., Hohlastos E., Riaz A., Kallini J., Gabr A., Ali R., Jenkins K., Karp J., Desai K. (2018). Prostate Artery Embolization for Lower Urinary Tract Symptoms Secondary to Benign Prostatic Hyperplasia: Results From a Prospective FDA-Approved Investigational Device Exemption Study. Urology.

[42] Tian W., Zhou C., Leng B., Shi H., Liu S. (2019). Prostatic Artery Embolization for Control of Gross Hematuria in Patients with Benign Prostatic Hyperplasia: A Single-Center Retrospective Study in 20 Patients. J. Vasc. Interv. Radiol..

[43] Wang M., Guo L., Duan F., Yuan K., Zhang G., Li K., Yan J., Wang Y., Kang H. (2016). Prostatic arterial embolization for the treatment of lower urinary tract symptoms caused by benign prostatic hyperplasia: A comparative study of medium- and large-volume prostates. BJU Int..

[44] Wang M.Q., Wang Y., Yan J.Y., Yuan K., Zhang G.D., Duan F., Li K. (2016). Prostatic artery embolization for the treatment of symptomatic benign prostatic hyperplasia in men ≥75 years: A prospective single-center study. World J. Urol..

[45] Yu S.C.H., Cho C.C.M., Hung E.H.Y., Chiu P.K.F., Yee C.H., Ng C.F. (2017). Prostate Artery Embolization for Complete Urinary Outflow Obstruction Due to Benign Prostatic Hypertrophy. Cardiovasc. Interv. Radiol..

[46] Yu S.C.H., Cho C.C.M., Hung E.H.Y., Zou J., Yuen B.T.Y., Shi L., Chiu P.K.F., Yee S.C.H., Ng A.C.F. (2019). Thickness-to-Height Ratio of Intravesical Prostatic Protrusion Predicts the Clinical Outcome and Morbidity of Prostatic Artery Embolization for Benign Prostatic Hyperplasia. J. Vasc. Interv. Radiol..

[47] Marik P.E., Taeb A.M. (2017). SIRS, qSOFA and new sepsis definition. J. Thorac. Dis..

[48] de Assis A.M., Moreira A.M., de Paula Rodrigues V.C., Yoshinaga E.M., Antunes A.A., Harward S.H., Srougi M., Carnevale F.C. (2015). Prostatic Artery Embolization for Treatment of Benign Prostatic Hyperplasia in Patients with Prostates > 90 g: A Prospective Single-Center Study. J. Vasc. Interv. Radiol..

[49] Cochran S.T., Barbaric Z.L., Lee J.J., Kashfian P. (1991). Percutaneous nephrostomy tube placement: An outpatient procedure?. Radiology.

[50] de la Motte L., Kehlet H., Vogt K., Nielsen C.H., Groenvall J.B., Nielsen H.B., Andersen A., Schroeder T.V., Lönn L. (2014). Preoperative Methylprednisolone Enhances Recovery After Endovascular Aortic Repair: A Randomized, Double-Blind, Placebo-Controlled Clinical Trial. Ann. Surg..

[51] Ogasawara S., Chiba T., Ooka Y., Kanogawa N., Motoyama T., Suzuki E., Tawada A., Nagai K., Nakagawa T., Sugawara T. (2018). A randomized placebo-controlled trial of prophylactic dexamethasone for transcatheter arterial chemoembolization. Hepatology.

[52] De Oliveira G.S., Almeida M.D., Benzon H.T., McCarthy R.J. (2011). Perioperative Single Dose Systemic Dexamethasone for Postoperative Pain: A Meta-analysis of Randomized Controlled Trials. Anesthesiology.

